# A critical role for the inward rectifying potassium channel Kir7.1 in oligodendrocytes of the mouse optic nerve

**DOI:** 10.1007/s00429-020-02043-4

**Published:** 2020-02-21

**Authors:** Maria Papanikolaou, Arthur M. Butt, Anthony Lewis

**Affiliations:** Institute of Biomedical and Biomolecular Sciences, School of Pharmacy and Biomedical Science, University of Portsmouth, Portsmouth, PO1 2DT UK

**Keywords:** Oligodendrocyte, Inward rectifying potassium channel, Potassium regulation, VU590, White matter

## Abstract

Inward rectifying potassium channels (Kir) are a large family of ion channels that play key roles in ion homeostasis in oligodendrocytes, the myelinating cells of the central nervous system (CNS). Prominent expression of Kir4.1 has been indicated in oligodendrocytes, but the extent of expression of other Kir subtypes is unclear. Here, we used qRT-PCR to determine expression of Kir channel transcripts in the mouse optic nerve, a white matter tract comprising myelinated axons and the glia that support them. A novel finding was the high relative expression of Kir7.1, comparable to that of Kir4.1, the main glial Kir channel. Significantly, Kir7.1 immunofluorescence labelling in optic nerve sections and in isolated cells was localised to oligodendrocyte somata. Kir7.1 are known as a K^+^ transporting channels and, using patch clamp electrophysiology and the Kir7.1 blocker VU590, we demonstrated Kir7.1 channels carry a significant proportion of the whole cell potassium conductance in oligodendrocytes isolated from mouse optic nerves. Notably, oligodendrocytes are highly susceptible to ischemia/hypoxia and this is due at least in part to disruption of ion homeostasis. A key finding of this study is that blockade of Kir7.1 with VU590 compromised oligodendrocyte cell integrity and compounds oligodendroglial loss in ischemia/hypoxia in the oxygen–glucose deprivation (OGD) model in isolated intact optic nerves. These data reveal Kir7.1 channels are molecularly and functionally expressed in oligodendrocytes and play an important role in determining oligodendrocyte survival and myelin integrity.

## Introduction

Oligodendrocytes myelinate axons in the central nervous system (CNS) and are essential for the rapid conduction of neural impulses. Bundles of myelinated axons form the white matter (WM), which interconnect the different regions of the CNS. Loss of oligodendrocytes and subsequently myelin has devastating effects on CNS function, such as occurs in the demyelinating disease Multiple Sclerosis (MS) as well as other neuropathologies including stroke and traumatic injury (Butt et al. 2014). Notably, hypoxia/ischemia is a key determining factor in all these pathologies, causing progressive depolarisation of the oligodendroglial membrane potential and ultimately resulting in cell death (Fern et al. 2014). Oligodendroglial membrane potential is largely determined by plasmalemmal inward rectifying potassium channels (Kir) (Neusch et al. 2001). There are seven subfamilies of Kir alpha subunits (Kir1–Kir7), each of which has multiple subfamily members, e.g. Kir4.1, Kir4.2, etc. Functional Kir channels are formed either through homomeric alpha subunit tetramerisation, or by heteromeric assembly, thereby increasing functional diversity (Hibino et al. 2010). In oligodendrocytes, a prominent role for Kir4.1 channels has been demonstrated in myelination and maintaining WM integrity under pathological conditions (Neusch et al. 2001; Bolton and Butt 2006; Schirmer et al. 2018). In addition, oligodendrocytes have been reported to express Kir5.1 and Kir2.1 as heteromers with Kir4.1 (Brasko et al. 2017; Brasko and Butt 2018). The most recently described Kir channel subtype is Kir7.1, which is encoded by the Kcnj13 gene and, like Kir4.1, is classified functionally as a K^+^ transporting Kir. However, in contrast to Kir4.1, Kir7.1 displays low sensitivity to barium and internal magnesium blockade, and to changes in external potassium concentration. Furthermore, Kir7.1 has not been reported to form heteromeric channels (Doring et al. 1998; Krapivinsky et al. 1998).

Kir7.1 is highly expressed in transporting epithelia, including the kidney, intestine, retinal pigment epithelium (RPE) and choroid plexus, where it is thought to function to recycle K^+^ across the cell membrane, providing the substrate K^+^ ions essential for transepithelial NaCl reabsorption by primary and secondary active transporters that is essential for osmotic water movement (Nakamura et al. 1999; Derst et al. 2001; Yang et al. 2003). Dysfunction of Kir7.1 induces degeneration of the RPE leading to the inherited eye pathologies Snowflake Vitreoretinal Degeneration (SVD) and Lebers Congenital Amaurosis (LCA) (Kumar and Pattnaik 2014). Moreover, recent studies utilising the well characterised Kir7.1 blocker, VU590, have revealed a novel role for Kir7.1 channels in regulating uterine excitability during pregnancy (McCloskey et al. 2014; Crankshaw et al. 2017) and in melanocortin signalling (Ghamari-Langroudi et al. 2015).

In the CNS, Kir7.1 was originally reported to be expressed principally in Purkinje neurons and pyramidal neurons in the hippocampus (Krapivinsky et al. 1998), but we have recently provided evidence of heterogeneous expression of Kir7.1 in glial cells in the brain (Papanikolaou et al. 2019). Here, we demonstrate expression of Kir7.1 in the mouse optic nerve, a typical CNS white tract. We show that oligodendrocytes express functional Kir7.1 using immunohistochemistry and patch-clamp electrophysiology, and using the well characterised Kir7.1 blocker VU590 we reveal a critical role for Kir7.1 channels in oligodendrocyte integrity.

## Materials and methods

### Experimental animals

All animals were killed by cervical dislocation, in accordance with regulations issued by the Home Office of the United Kingdom under the Animals (Scientific Procedures) Act, 1986. The animals used were C57BL6/10 wild type strains or transgenic mouse strains; PLP-DsRed (kindly provided by Frank Kirchhoff, University of Saarland, Germany) and SOX10-eGFP (kindly provided by William D Richardson, UCL, UK) in which the fluorescent reporters DsRed and eGFP are driven by the oligodendroglial genes PLP1 and Sox10, respectively (Hirrlinger et al. 2005; Matsuoka et al. 2005; Kessaris et al. 2006). Brains and optic nerves were dissected free and either treated for qRT-PCR or placed immediately in in 4% paraformaldehyde (PFA) for immunohistochemistry, in dissecting medium for explant cultures, or artificial cerebrospinal fluid (*a*CSF) for Oxygen and Glucose Deprivation (OGD) experiments.

### qRT-PCR

Optic nerves were isolated and RNA extraction was performed maintaining strict RNase-free and sterile conditions throughout using published protocols (Papanikolaou et al. 2017). RNA was processed using an RNeasy Micro kit (Qiagen) and converted to single stranded cDNA using the RT2 First Strand Kit (Qiagen) following manufacturer’s instructions. The quantity of RNA that was transcribed was the same for all samples (500 ng). cDNA libraries were prepared from total RNA extracted from ten pooled optic nerves from postnatal mice (aged postnatal day P9–12), and adult mice (aged P30–40), and analyses were run in triplicate. SYBR Green qPCR Mastermix (Qiagen) was mixed with cDNA and ultra-pure water (Ambion) and 25 μl was pipetted in each well of the 96 well-plate arrays for the Lightcycler 96 (Roche), using the Mouse Neuronal Ion Channels RT2 Profiler™ qPCR array and a custom RT^2^ Profiler™ qPCR array for additional channels not included in the neuronal array, namely Kir4.1 (Sabiosciences, Qiagen). Relative gene expression was determined using the ^ΔΔ^-CT method versus GAPDH, which was identified as the most appropriate housekeeping gene using the Normfinder algorithm and the standard deviation (SD) method. Gene expression data are presented as mean ± SEM, and samples compared for significance using one-way ANOVA to investigate differences in expression of all genes within an age range, and unpaired *t* tests for developmental differences of individual genes in Prism 6.0 (Graphpad).

### Reverse transcription PCR

RNA extraction was performed on isolated optic nerves and whole brain as described for qRT-PCR. First strand cDNA synthesis was carried out using the Transcriptor First Strand cDNA Synthesis Kit (Roche, Burgess Hill, West Sussex, UK). High quality cDNA libraries of the whole mouse brain and optic nerve were used in downstream Polymerase Chain Reactions (PCR) with primers for Kir7.1. The PCR reaction volume was 50 µl (14 µl ddH_2_O; 1 µl cDNA (1 µg); 25 µl DreamTaq (2×); 5 µl Forward Primer (10 µM); 5 µl Reverse Primer (10 µM)). The primers were designed using the National Center for Biotechnology Information (NCBI) Primer-BLAST tool (https://www.ncbi.nlm.nih.gov/tools/primer-blast/) and synthesised by Invitrogen (Kir7.1 Forward Primer: cacatcaccagcttcacagc, Kir7.1 Reverse Primer: ggtttgccatctttgtgagc). The product amplified by the primers is a 251 bp amplicon spanning exons 2 and 3 of the mouse KCNJ13 gene.

### Western blot

Protein was extracted from mouse optic nerve and cerebellum and western blots were performed using published protocols (Brasko et al. 2017). In brief, tissue was homogenised in RIPA buffer 1× complete mini protease inhibitor cocktail (Roche; Burgess Hill, UK) using a Bertin Minilys. Samples were centrifuged at 4 °C, at high speed (14,000 rpm) for 15 min and supernatant was transferred in clean eppendorfs. Quantification of protein concentration was carried out using the bicinchoninic acid assay (Sigma) with a standard bovine serum albumin (BSA) concentration curve and UV spectrophotometer (POLAR star OPTIMA, BMG LabTech; Ortenberg, Germany). Samples were mixed with Laemmli buffer, heated at 70 °C for 10 min with β-mercaptoethanol and 60 μg of protein per lane was loaded for 10% acrylamide sodium dodecyl sulfate polyacrylamide gel electrophoresis (SDS-PAGE). Proteins were then electrophoretically transferred to a polyvinyllidene difluoride membrane (Amersham) which was then incubated in blocking solution 5% *w*/*v* dried milk in TBS (150 mM NaCl, 10 mM Tris, pH7.4 with 1% *w*/*v* Tween 20). Incubation in rabbit anti-Kir7.1 antibody at 1:200 (Alomone) was carried out overnight at 4 °C, and following washes, the secondary antibody horseradish peroxidase-conjugated goat anti-rabbit (Agilent; Santa Clara, CA, USA) was added at 1:5000 for 2 h at RT; controls were preincubated with the competitive peptide from which the Kir7.1 antibody was raised. Extensive washing of the membranes in TBS with 1% *w*/*v* Tween 20 was performed after each incubation and immunocomplexes were detected using the Luminata Forte chemiluminescence HRP detection reagent (Millipore). Finally, mouse β-actin (1:3000, Sigma) incubation for 30 min was used as a positive control, followed by 1 h incubation with HRP-conjugated goat anti-mouse (1:5000, Agilent).

### Optic nerve explant cultures

Optic nerve explant cultures were prepared as previously described (Brasko et al. 2017). Briefly, optic nerves from P7 to P12 mice were placed into dissecting medium consisting of high glucose Dulbecco’s Modified Eagle Medium (Sigma; Irvine, UK) containing 10% Fetal Calf Serum (Fisher; Loughborough, UK), l-Glutamine (Sigma) and 0.1% Gentamycin (Fisher). Nerves were finely chopped with a scalpel blade and triturated with pipettes of decreasing diameter. The solution was then pipetted onto poly-d-lysine/matrigel (Fisher) coated coverslips and after 24 h, was replaced with a low serum (0.5%) modified Bottenstein and Sato (B&S) medium (Bottenstein and Sato 1979). Explant cultures were used for immunolabelling or patch clamp after 10–12 days in vitro (DIV).

### Immunolabelling

Optic nerves were fixed with 4% PFA for 1–2 h, washed in PBS and then cryoprotected in 10, 20 and 30% sucrose at 4 °C for 48–72 h, prior to embedding in Cryo-M-Bed (Bright Instruments; Luton, UK) for storage at − 80 °C. Longitudinal optic nerve sections (14 μm) were cut with a Leica CM3050 S cryostat at − 21 °C and transferred onto Polysine® coated slides (Fisher). For explant cultures, coverslips were fixed for 20 min in 1% PFA and washed thoroughly in PBS prior to use. After this, optic nerve sections and cell cultures were treated the same. Following washes in PBS for 30 min at room temperature (RT), a blocking stage was performed using 5% normal goat serum (NGS) in PBS for 1 h at RT; where primary antibodies targeted an intracellular epitope, Triton X-100 (Sigma) was included in the blocking solution (0.1% for sections and 0.01% for cultured cells). The primary antibody rabbit anti-Kir7.1 (Alomone, Israel) was diluted 1:300 in blocking solution and tissues/cells incubated overnight at 4 °C. Samples were then washed three times in PBS and incubated with the appropriate secondary antibodies conjugated with Alexafluor 488, 568 or 649 (1:400, Fisher) as well as with the DNA dye Hoechst Blue (Fisher 1:1000). Controls were carried out, in which sections/cells were incubated in primary antibody that was previously preabsorbed with antigen peptide. Following immunolabelling, coverslips/sections were mounted with Fluoromount-G® (Southern Biotech; Birmingham, AL, USA). Images were acquired using a Zeiss Axiovert LSM710 VIS405 confocal microscope, using multichannel sequential scanning, narrow bandwidths, and minimal laser power and gain to prevent cross-talk between the channels. Colocalisation analysis was performed using Volocity 6.3 (Perkin Elmer; Beaconsfield, UK), as described previously (Brasko et al. 2017).

### Whole cell patch clamp

Patch-clamp recordings were performed on oligodendrocytes from optic nerve explant cultures at 10–12 DIV, as described previously (Hawkins and Butt 2013). Patch electrodes (2–8 MΩ) were backfilled with intracellular solution (ICS) containing (in mM): potassium acetate 90; KCl 20; HEPES 40; MgCl_2_ 3; EGTA 3; CaCl_2_ 1; pH 7.4; plus 3 mM Na_2_ATP, to block K_ATP_. The extracellular solution (ECS) was aCSF; NaCl 106; KCl 30; CaCl_2_ 2.24; NaH_2_PO_4_ 1.2; MgCl_2_ 1; HEPES 8.55. The liquid junction potential was calculated to be 7.5 mV (pCLAMP10 software, Molecular Devices) and IV curves were not corrected for this. Oligodendrocytes used for recording had cell capacitance ranging from 10 to 50 pF and recordings with series resistance larger than 3 × pipette resistance or holding current of > 100 nA were discarded. Cells were held at − 50 mV in voltage clamp and 10 mV steps of 500 msec duration were applied from − 130 to 60 mV. Cells were subjected to voltage step protocols first in normal ECS, then repeated in the presence of the specific Kir7.1 channel small-molecule inhibitor VU590 (7,13-bis(4-nitrobenzyl)-1,4,10-trioxa-7,13-diazacyclopentadecane dihydrochloride; 20 μM, Tocris; Bristol, UK) (Lewis et al. 2009; Bhave et al. 2011), and finally in VU590 plus the broad spectrum Kir blocker Ba^2+^ (100 µM). Currents were recorded using a CV 203Bu Headstage and an Axo-patch 200B amplifier, with frequencies > 1 kHz filtered, and digitisation achieved (sampling at 5 kHz) through a DigiData 1440A interface (Molecular Devices; Wokingham, UK). The pCLAMP10 software package (Molecular Devices) was used for data acquisition and analysis, and significance between peak currents determined by *t* tests, using Prism 7.0 (Graphpad; La Jolla, CA, USA).

### Oxygen and glucose deprivation

Optic nerves from P12–14 transgenic Sox10-eGFP mice were isolated intact and immediately placed in aCSF for 30 min at 37 °C in a normoxic incubator (95% air/5% CO_2_) and allowed to stabilise. Control groups were incubated for a further 1 h in normal aCSF containing glucose with 5% CO_2_/95% air (Oxygen and Glucose Normal; OGN) without the addition of pharmacological modulators. Oxygen–glucose deprivation (OGD) was achieved using the method of Fern and colleagues (2005), by incubating nerves for 1 h at 37 °C in glucose-free aCSF (osmolarity was maintained by replacing glucose with sucrose), and switching the chamber atmosphere to 5% CO_2_/95% N_2_. The Kir7.1 channel blocker VU590 was added to the aCSF solution of test groups during OGN and OGD; due to the restricted permeability of the pia in the intact optic nerve, VU590 was used at a concentration of 100 μM. At the end of experiments, nerves were fixed for 1 h in 4% PFA, whole-mounted with FluoroMount and examined using a Zeiss Axiovert LSM710 VIS405 confocal microscope and maintaining all variables constant between images. The total number of cells was counted in five fields of view (FOV) along the length of each optic nerve and the optic nerve means were used for statistical analysis. The FOV comprised a constant volume of 20 × 20 µm in the* x–y*-plane and 15 × 1 µm optical sections in the* z*-plane, commencing 15 µm below the pial surface. Data are presented as mean ± SEM (*n* ≥ 4, where *n* represents the number of nerves), and significance was determined by one-way ANOVA followed by Tukey’s multiple comparison post-hoc analysis, using Graphpad Prism 7.0.

## Results

### Kir7.1 is prominently expressed in CNS white matter

**Fig. 1:**
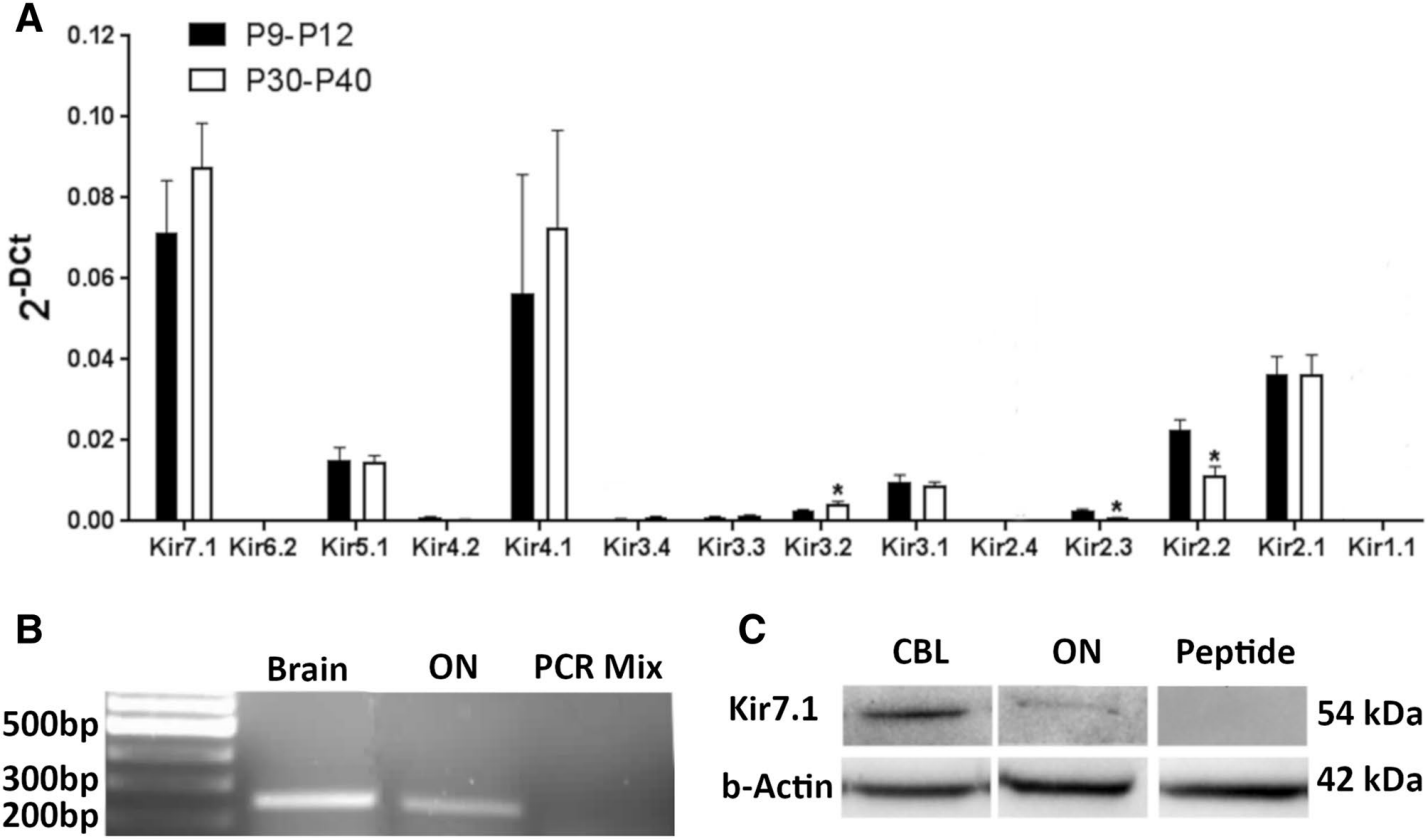
Kir7.1 expression in the mouse optic nerve. **a** qRT-PCR analysis of Kir channels in acutely isolated optic nerves from adolescent (P9–12) and young adult (P30–40) mice; *p < 0.01, one-way ANOVA with subsequent multiple comparisons; qRT-PCRs were ran in triplicate for each age group, with *n* = 10 optic nerves per run (*n* = 5 animals per qRT-PCR). **b**, **c** Confirmation of Kir7.1 expression in the optic nerve by comparison with brain, by RT-PCR (**b**) and Western blot (**c**), with detection of the expected Kir7.1 mRNA product at 251 bp (**b**) and protein at 54 kDa (**c**); no bands were detected in negative controls, in the absence of cDNA in the reaction mix for RT-PCR (**b**) and pre-incubation with the blocking peptide for western blots (**c**). Due to very similar MW of the proteins, the same samples were run in parallel and under identical conditions on different western blots, using β-actin as a positive control to demonstrate the presence of Kir7.1 protein

White matter integrity is essential for CNS function and depends greatly on the homeostatic function of glial Kir channels (Schirmer et al. 2018). However, with the exception of the predominant glial potassium channel Kir4.1 (Kcnj10), the expression profile of Kir subtypes expressed by glial cells is unclear (Butt and Kalsi 2006). To address this, we performed qRT-PCR analysis of the mouse optic nerve, a model CNS white matter tract that contains significant numbers of oligodendrocytes and astrocytes. Given that the optic nerve tract contains axons but not neuronal somata, the transcriptome in whole optic nerve extracts represents that of glial mRNA (Salter and Fern 2005). Gene transcripts were detected using the Mouse Neuronal Ion Channels RT2 Profiler™ qPCR array (Kcnj1, Kcnj2, Kcnj3, Kcnj4, Kcnj5, Kcnj6, Kcnj9, Kcnj11, Kcnj12, Kcnj13, Kcnj14, Kcnj15, Kcnj16) and a custom RT^2^ Profiler™ qPCR array for Kir4.1 (Kcnj10). Transcript levels were normalized against the housekeeping gene GAPDH by the comparative ^ΔΔ^-CT method (Fig. 1a) (Papanikolaou et al. 2017). To examine potential developmental changes in the Kir profile, optic nerves were analysed from postnatal mice (postnatal day (P)9–12) and young adult (P30–40). Data were from ten pooled optic nerves per age group, run in triplicate, with data expressed as mean ± SEM (*n* = 3). A novel finding was that relative transcript levels for Kir7.1 (Kcnj13) were comparable to Kir4.1 (Kcnj10), which is generally recognised as the main glial potassium channel (Butt and Kalsi 2006), and has been shown previously to be highly expressed by optic nerve astrocytes and oligodendrocytes (Kalsi et al. 2004; Bay and Butt 2012; Brasko et al. 2017). There appeared to be no developmental regulation of Kir4.1 or Kir7.1 expression and relative transcript levels for these two genes were significantly greater than for any other Kir subtype gene (****p < 0.0001, one-way ANOVA). However, Kir2.1 (Kcnj2) and Kir5.1 (Kcnj16) were also prominent in both age groups, consistent with our recent studies demonstrating their co-expression with Kir4.1 in optic nerve glia (Brasko et al. 2017; Brasko and Butt 2018). Other subtypes including Kir2.2 (Kcnj12), Kir3.1 (Kcnj3) and Kir3.2 (Kcnj6) were expressed at lower levels, whilst Kir2.3 (Kcnj4), Kir3.3 (Kcnj9), Kir3.4 (Kcnj5) and Kir6.2 (Kcnj11) were negligible, and Kir1.1 (Kcnj1) encoding the renal outer medullary K^+^ channel (ROMK) channel was absent (Fig. 1a), consistent with its almost exclusive expression in kidney (Lewis et al. 2009; Kharade et al. 2017). Expression of Kir7.1 in the optic nerve was confirmed using RT-PCR (Fig. 1b) and western blot (Fig. 1c), with predicted bands at 251 bp for mRNA (Fig. 1b) and 54 kDa for protein (Fig. 1c) (Krapivinsky et al. 1998; Pattnaik et al. 2013); positive bands were absent in negative controls, in the absence of cDNA in the reaction mix for RT-PCR (Fig. 1b) and in the presence of the competitive peptide for western blot (Fig. 1d).

### Kir7.1 channels are expressed by oligodendrocytes

**Fig. 2:**
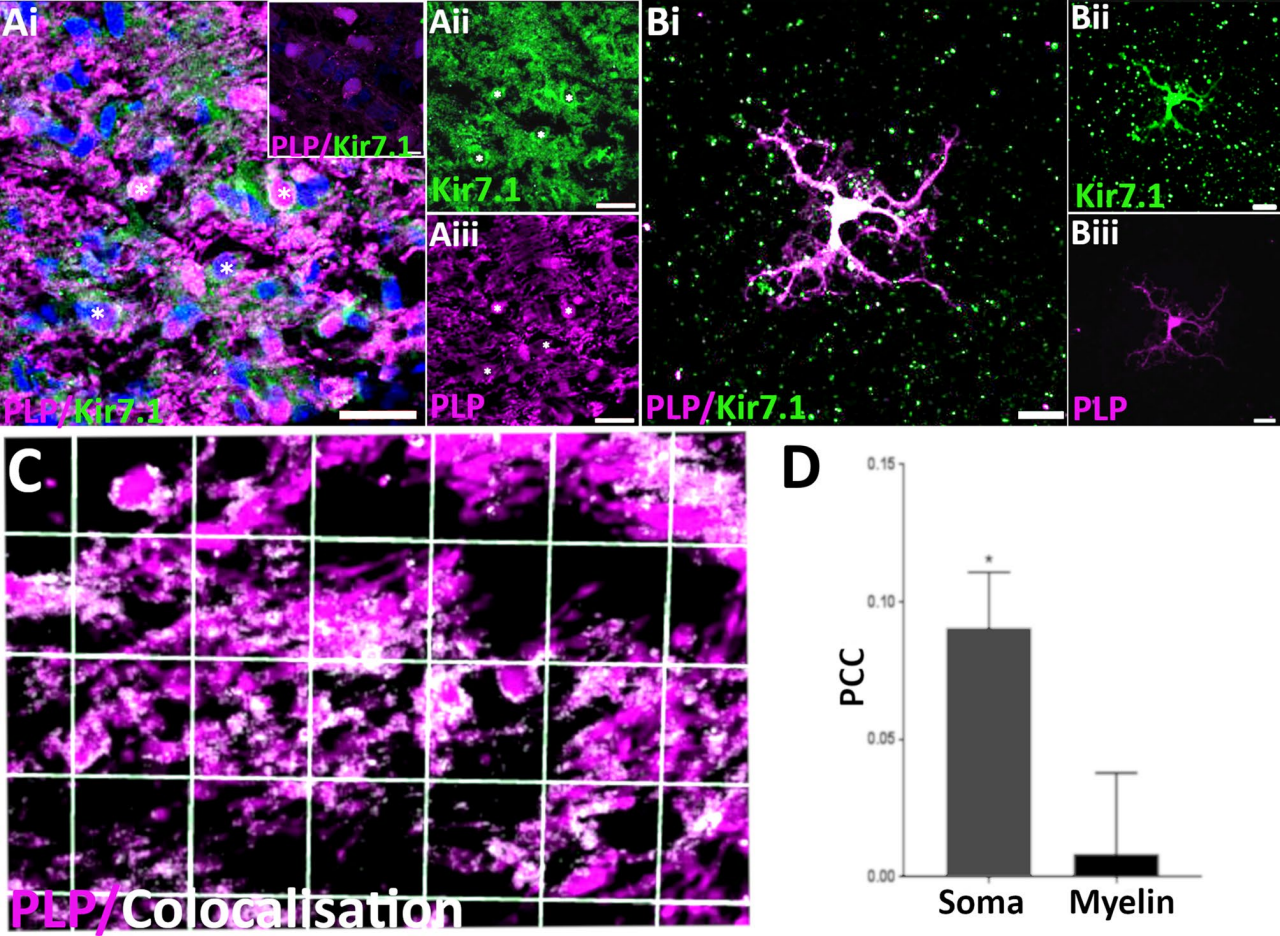
Expression of Kir7.1 in optic nerve oligodendrocytes. **a** Optic nerve sections from PLP-DsRed reporter mice to identify oligodendrocytes (here shown in magenta), showing oligodendrocyte somata immunopositive for Kir7.1 (some indicated by asterisks), while there is less immunostaining in the myelinated fascicles (**ai**, overlay image; **aii**, **aiii**, individual channels; **ai** inset, negative control pre-incubated in blocking peptide. **b** Oligodendrocytes from optic nerve explant cultures immunostained for Kir7.1; **bi** illustrates the overlay, where co-labelling appears white; **bii** and **biii** are the individual channels for Kir7.1 and the PLP-DsRed reporter respectively. **c** Colocalisation analysis of Kir7.1 and PLP-DsRed in situ in the P15 mouse optic nerve (white indicates voxels in which the magenta and green channels are expressed at the same level). **d** Mean thresholded Pearson’s correlation coefficient (PCC), showing significantly greater colocalisation between Kir7.1 and PLP-DsRed in oligodendroglial somata than in myelin (*p < 0.05, unpaired *t* test; Mean ± SEM, *n* = 32 cells from three animals). Scale bars = 25 μm

We performed immunohistochemistry to examine cellular expression of Kir7.1 in the optic nerve from PLP-DsRed reporter mice, in which the myelin gene proteolipid protein (PLP) drives expression of the reporter DsRed (Fig. 2a; the red colour of DsRed was changed to magenta to comply with the principles of Colour Universal Design (Ichihara et al. 2008)). In optic nerve sections, immunostaining for the Kir7.1 protein showed a broad, overlapping profile with PLP reporter fluorescence. Immunoreactivity was absent in negative controls following pre-absorption with the blocking peptide (inset in Fig. 2ai). Oligodendroglial expression of Kir7.1 was confirmed unequivocally in optic nerve explant cultures (Fig. 2b). To examine the relative expression levels of Kir7.1 on the cell somata compared to the myelin sheaths, a colocalization channel was generated to identify the individual voxels in which the red (PLP-DsRed, visualised as magenta) and green (Kir7.1 immunostaining) channels overlap with the same intensity (Fig. 2c), using the thresholded Pearson’s Correlation Co-efficient (PCC), as described previously (Brasko et al. 2017). The results demonstrated significant co-localization of Kir7.1 with PLP-DsRed in oligodendroglial somata (Fig. 2c, co-localization appears white) and the thresholded PCC confirms Kir7.1 was significantly greater on the cell somata than in the myelinated fascicles (Fig. 2d).

### Kir7.1 channels generate a functional potassium conductance in oligodendrocytes

**Fig. 3:**
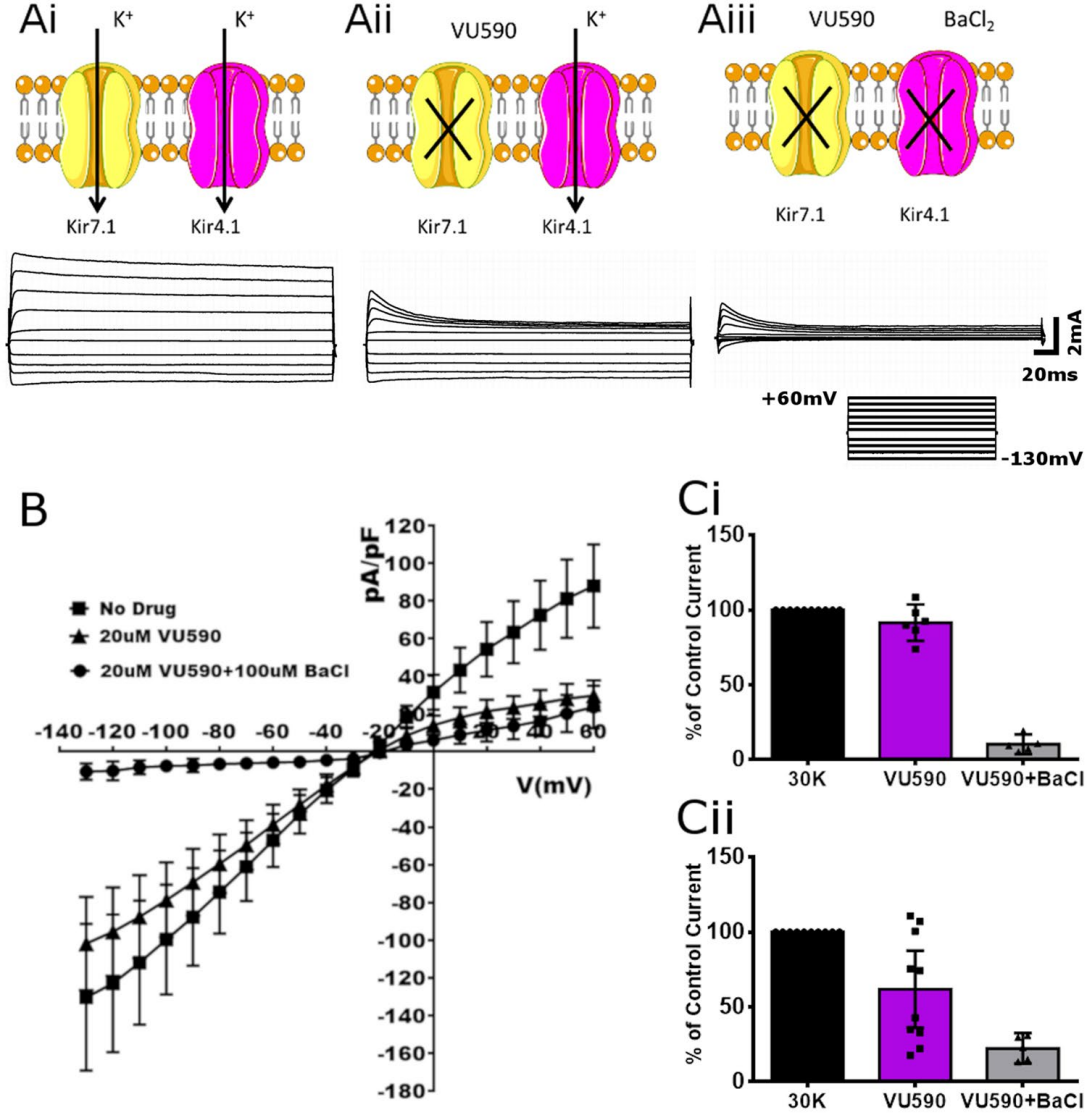
Kir7.1 mediate potassium currents in optic nerve oligodendrocytes. Whole-cell patch clamp analysis in oligodendrocytes from optic nerve explant cultures. **a** VU590-sensitive Kir7.1-like currents. 10 mV depolarising voltage steps from − 130 to + 60 mV were applied in high extracellular K^+^ in the absence of any pharmacological agents (**ai**), in the presence of the specific Kir7.1 blocker VU590 (20 µM) (**aii**) or VU590 and the Kir4.1 blocker BaCl_2_ (100 µM) (**aiii**). **b, c** The results (*n* = 4) are plotted as I–V relations before and after exposure to Kir blockers (**b**), and peak current density is expressed relative to the no drug condition in the same cells, illustrating a decrease in the presence of the blockers at − 130 mV (**ci**) as well as at + 60 mV (**cii**) (***p  ≤ 0.001, ****p  ≤ 0.0001; one-way ANOVA with Tukey’s multiple comparison’s test, *n* ≥ 4)

These data clearly demonstrate that oligodendrocytes express the Kir7.1 channel protein. The next step was to examine their functionality at the plasma membrane. Therefore, we performed whole cell patch clamp analysis of membrane currents of cultures of optic nerve oligodendrocytes, as previously described (Hawkins and Butt 2013), and used the non-selective Kir blocker Ba^2+^ and the selective Kir7.1 blocker VU590 to isolate the Kir7.1 potassium conductance (Fig. 3). Under basal conditions, the* I–V* relations indicated the E_rev_ was approximately − 20 mV, which when taking into account the liquid junction potential offset of 7.5 mV and the likelihood of cell membrane permeability to other ions, such as Na^+^, Cl^−^ and Ca^2+^ approximates that predicted by the Nernst equation for potassium, − 33 mV (Fig. 3b). Oligodendrocytes express Kir4.1 that are completely blocked by Ba^2+^ (Bay and Butt 2012; Brasko et al. 2017), but are not blocked by VU590 (Lewis et al. 2009). Conversely, Kir7.1 are less sensitive to Ba^2+^ and are completely inhibited by VU590, which has proven a powerful pharmacological tool for isolating Kir7.1 function in uterine smooth muscle and hypothalamic neurones (McCloskey et al. 2014; Ghamari-Langroudi et al. 2015; Crankshaw et al. 2017); Kir1.1 channels are also inhibited by VU590 (Lewis et al. 2009), but the qPCR indicates they are not expressed by optic nerve glia (Fig. 1a). To determine the role of Kir7.1 on oligodendroglial potassium currents, whole cell oligodendrocyte currents were recorded in 30 mM extracellular potassium ([K^+^]_o_), firstly in the absence of any pharmacological blockers (Fig. 3ai). Addition of 20 µM VU590 blocked a significant portion of oligodendroglial steady-state outward and inward currents (Fig. 3aii). Co-addition of 20 µM VU590 and 100 µM BaCl_2_ resulted in an almost complete blockade of oligodendroglial steady-state currents (Fig. 3aiii), consistent with the functional predominance of Ba^2+^-sensitive Kir4.1 channel currents (Neusch et al. 2001). Analysis of mean current–voltage relationships (Fig. 3b), peak inward currents at − 130 mV (Fig. 3ci) and peak outward currents at + 60 mV (Fig. 3cii) demonstrated that over 60% of the outward current was VU590-sensitive, with no further blockade in the presence of Ba^2+^. This is in contrast to the current at − 130 mV where 15% of the inward current was VU590-sensitive with a large proportion of the remaining current Ba^2+^-sensitive (Fig. 3cii). The results demonstrate Kir7.1 channels are responsible for a significant K^+^ conductance in oligodendrocytes.

### Kir7.1 are cytoprotective for oligodendrocytes

**Fig. 4:**
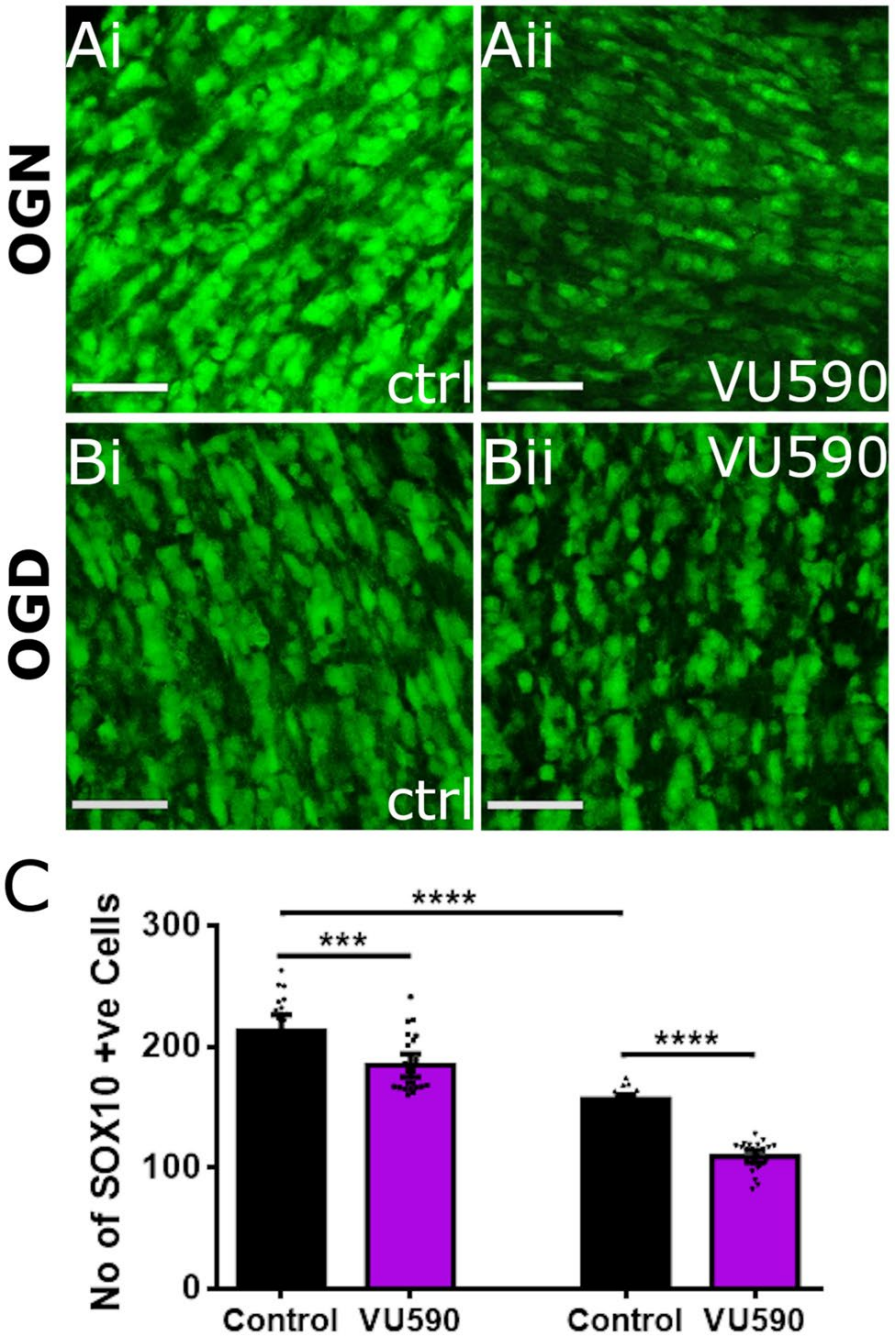
Kir7.1 channels are important for oligodendrocyte survival. Optic nerves from P12–P14 SOX10-eGFP mice were used to identify somata of oligodendrocyte lineage cells (OL), exposed for 1 h to normal oxygen and glucose (OGN) or oxygen and glucose deprivation (OGD) conditions, in the presence or absence of the specific Kir7.1 inhibitor VU590 (100 µM). **a**, **b** Representative images of optic nerves from SOX10-eGFP reporter mice incubated in OGN (**ai**) and OGD (**bi**) and in OGN + VU590 (**aii**) and OGD + VU590 (**bii**). **c** Quantification of the number of SOX10-eGFP positive cells (mean ± SEM; *n* = 4 nerves per group) revealed a dependence of OL cell survival on Kir7.1. It is also apparent that there is significant disruption and loss of OL cells during OGD compared to OGN, which was exacerbated by VU590 (***p < 0.001; ****p < 0.0001; one-way ANOVA with Tukey’s Multiple Comparisons test). Scale Bars = 50 μm

Kir have a critical role in ion and water homeostasis, which is essential for oligodendrocyte function and integrity (Neusch et al. 2001; Bolton and Butt 2006). To determine the importance of Kir7.1 in oligodendrocytes in the mouse optic nerve, we examined the impact of the Kir7.1 inhibitor VU590. Optic nerves from transgenic reporter mouse were used, in which the oligodendroglial Sox10 gene drives expression of enhanced green fluorescence (eGFP) to identify all cells of the oligodendroglial lineage (Fig. 4). Control optic nerves were incubated in normoxic conditions (95% air/5% CO_2_ and normal aCSF (10 mM glucose) also referred to as “Oxygen and Glucose Normal” (OGN) for 60 min in the absence of pharmacological agents or in the presence of VU590. (Fig. 4ai). Blockade of Kir7.1 channels with VU590 resulted in a significant loss of oligodendrocytes under normoxic conditions (Fig. 4aii, c). Similarly, exposure of optic nerves to compromised oxygen and glucose levels (5% CO_2_/95% N_2_) also referred to as “Oxygen and Glucose Deprivation” (OGD) for 60 min, as might be expected during hypoxia/ischaemia, resulted in a marked loss of oligodendrocyte lineage cells (Fig. 4bi, c), consistent with previous observations (Hawkins and Butt 2013). Loss of SOX10 positive cells in OGD was significantly greater following inhibition of Kir7.1 using VU590 (Fig. 4bii, c; *p* < 0.001, one-way ANOVA with Tukey’s multiple comparisons test).

## Discussion

**Fig. 5:**
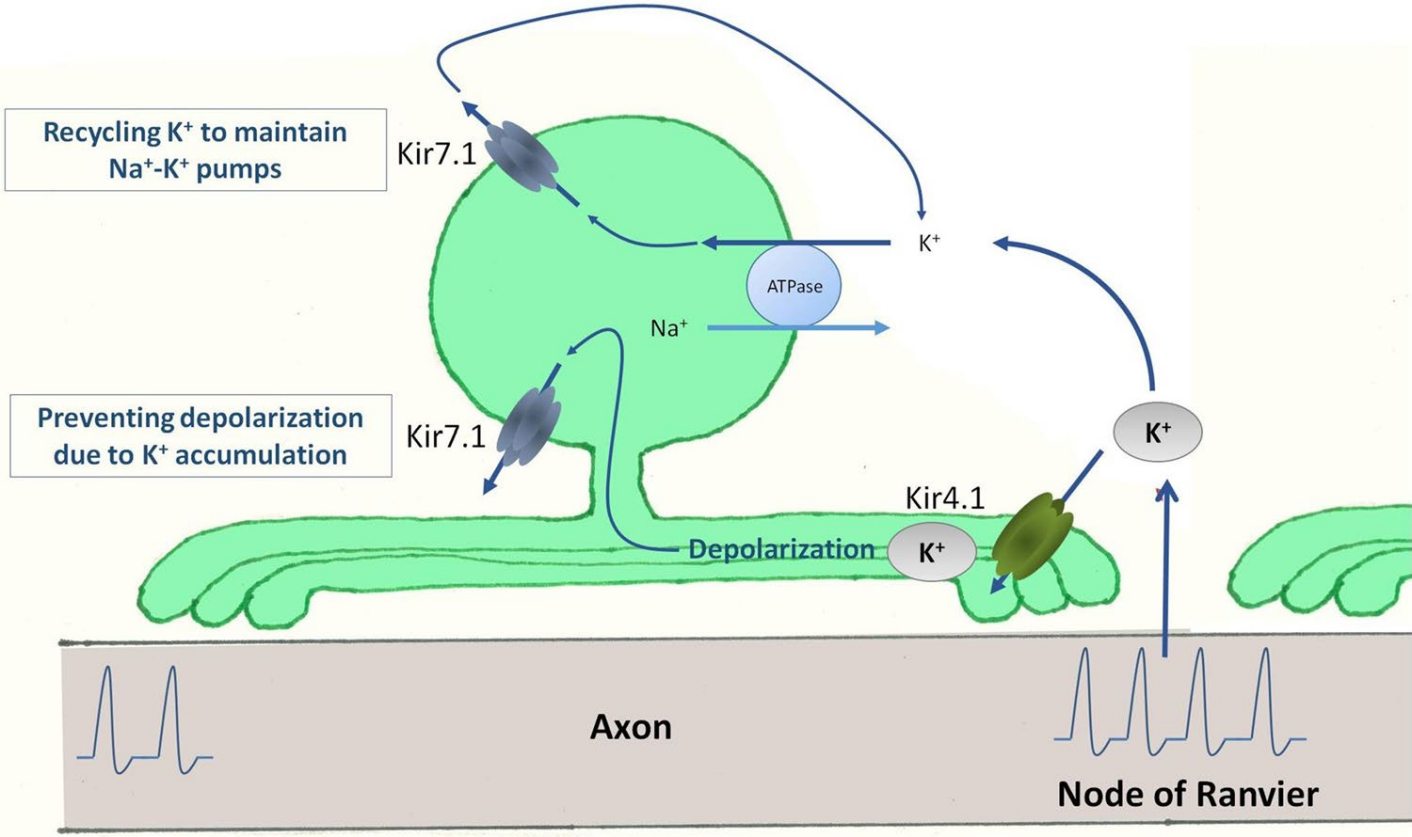
Model of Kir7.1 function in oligodendrocytes. Axonal action potential propagation results in continuous K^+^ release into the extracellular space, which is taken up by oligodendrocytes through Kir4.1 and by the activity of Na^+^–K^+^ pumps. Potassium is redistributed through Kir7.1, which acts to protect the cell in the face of these depolarizing influences and to recycle K^+^ that is essential for maintaining Na^+^–K^+^ pump activity. Blockade of Kir7.1 with VU590 results in oligodendrocyte demise and this is exacerbated in ischaemia, where Na^+^–K^+^ pumps are compromised

Inward rectifier potassium (Kir) channels have an essential physiological role in glial ion and water homeostasis (Butt and Kalsi 2006). Studies involving the genetic ablation of the Kir4.1 gene provide strong evidence for a key functional role for Kir4.1 channels in oligodendrocytes and white matter integrity (Neusch et al. 2001; Schirmer et al. 2018). Recent studies provide evidence that oligodendrocytes also express other Kir channel subtypes, including Kir5.1, Kir2.1 and Kir7.1 (Papanikolaou et al. 2019; Brasko et al. 2017; Brasko and Butt 2018). In the present study, using qRT-PCR, we discovered that transcripts for Kir7.1 are expressed at equivalent levels as Kir4.1 in the optic nerve, a typical white matter tract. This prompted us to examine Kir7.1 expression in oligodendrocytes using immunohistochemistry and patch-clamp electrophysiology. The results demonstrate Kir7.1 are prominently expressed by oligodendrocytes. Significantly, we show that blocking Kir7.1 with the specific antagonist VU590 causes a loss of oligodendrocytes. Moreover, oligodendrocytes are highly susceptible to ischemia/hypoxia and we show that blocking Kir7.1 exacerbates oligodendrocyte demise in the oxygen–glucose deprivation (OGD) model of ischemia. The results provide the first evidence of a critical role for Kir7.1 in maintaining oligodendrocyte integrity in the face of depolarizing stress (Fig. 5).

A key finding of this study is that relative transcript levels for Kir7.1 (Kcnj13) and Kir4.1 (Kcnj10) are comparable in the optic nerve. This finding is suggestive of an important physiological role for Kir7.1 in white matter glia, since Kir4.1 are considered the primary glial potassium channel (Butt and Kalsi 2006). Immunostaining demonstrated Kir7.1 protein expression in oligodendrocytes and myelin, using antibodies that have been validated in multiple tissues (Nakamura et al. 1999; Derst et al. 2001; Yang et al. 2003). Notably, blockade of Kir7.1 channel currents with VU590 resulted in the loss of oligodendrocytes and significantly augmented oligodendrocyte loss in ischemia/hypoxia. The results support an important role for Kir7.1 channels in maintaining the integrity of oligodendrocytes and myelin, particularly in the face of the depolarizing effects of an ischaemic insult. Under these conditions, the ability of the cell to produce ATP to fuel Na^+^–K^+^ active transport mechanism is compromised, resulting in a run-down of the resting membrane potential (Lipton 1999; Tekkok et al. 2007).

In conclusion, our study demonstrates functional expression of Kir7.1 in oligodendrocytes and supports a novel role for Kir7.1 in maintaining oligodendroglial integrity. Based on our findings, we posit that in the face of depolarizing influences efflux of potassium through Kir7.1 channels will act as a ‘closed loop’ to help maintain ion and water homeostasis. An equivalent function for Kir7.1 has been demonstrated in the RPE and kidney tubules, where Kir7.1 channels are co-localised with Na^+^/K^+^-ATPase (Ookata et al. 2000; Yang et al. 2003), and loss of Kir7.1 function results in RPE degeneration (Roman et al. 2018). In RPE, Kir7.1 channels have an important role in the regulation of the subretinal space volume by facilitating K^+^ ion efflux from the cells when [K^+^]_o_ is low, i.e. upon illumination and activation of retinal photoreceptor cells. Moreover, due to its weak inward rectification, Kir7.1 provides a means for K^+^ efflux that balances the counter influx of K^+^ ions occurring via Na^+^–K^+^ pumps and contributing to their continued functional efficiency, a process termed “K^+^ recycling” (Wimmers et al. 2007). In the optic nerve and other white matter tracts, axonal action potential propagation results in continuous K^+^ release into the extracellular space, which is taken up by oligodendrocytes through Kir4.1 and by the activity of Na^+^–K^+^ pumps. The redistribution of K^+^ via Kir7.1 will act to protect the cell in the face of these depolarizing influences and enable recycling of K^+^ which is essential for maintaining Na^+^–K^+^ pump activity (Fig. 5). Thus, blockade of Kir7.1 with VU590 results in oligodendrocyte demise and this is exacerbated in ischaemia, where Na^+^–K^+^ pumps are compromised. In summary, the results demonstrate a novel role for Kir7.1 in oligodendrocytes, which is critical for white matter physiology and pathology, including ischemia, traumatic injury and multiple sclerosis (Butt 2011; Fern et al. 2014; Rivera et al. 2016).
